# Bifidobacteria-Host Interactions—An Update on Colonisation Factors

**DOI:** 10.1155/2014/960826

**Published:** 2014-09-11

**Authors:** Verena Grimm, Christina Westermann, Christian U. Riedel

**Affiliations:** Institute of Microbiology and Biotechnology, University of Ulm, 89068 Ulm, Germany

## Abstract

Bifidobacteria are one of the predominant bacterial groups of the human intestinal microbiota and have important functional properties making them interesting for the food and dairy industries. Numerous *in vitro* and preclinical studies have shown beneficial effects of particular bifidobacterial strains or strain combinations on various health parameters of their hosts. This indicates the potential of bifidobacteria in alternative or supplementary therapeutic approaches in a number of diseased states. Based on these observations, bifidobacteria have attracted considerable interest by the food, dairy, and pharmaceutical industries and they are widely used as so-called probiotics. As a consequence of the rapidly increasing number of available bifidobacterial genome sequences and their analysis, there has been substantial progress in the identification of bifidobacterial structures involved in colonisation of and interaction with the host. With the present review, we aim to provide an update on the current knowledge on the mechanisms by which bifidobacteria colonise their hosts and exert health promoting effects.

## 1. Introduction

### 1.1. Host Colonisation by Bifidobacteria

On a cellular basis, humans can be regarded as superorganisms. As a rough approximation, these super-organisms consist of 90% microbial cells with the vast majority of the microbial diversity being located in the human gastrointestinal tract (GIT) [1]. The development and composition of a normal GIT microbiota is crucial for establishing and maintaining human health and well-being [2–4]. It is generally accepted that, before birth, the intrauterine environment and thus the GIT of the unborn foetus are sterile [4]. During delivery, newborns acquire microorganisms from their mothers faecal, vaginal, and skin microbiota. Interestingly, considerable numbers of bifidobacteria and other components of the infant intestinal microbiota were also isolated from human breast milk [5, 6]. Some of the strains recovered in the mother's milk were identical to those detected in the faecal samples of the infant [7] suggesting that human milk might contribute to the establishment and development of the intestinal microbiota of children.

The succession of colonisation follows more or less a classical pattern with facultative anaerobes such as* Escherichia coli* or* Enterococcus sp*. dominating for the first hours or days. Once these organisms have consumed the residual oxygen in the GIT, strictly anaerobic bacteria including* Bifidobacterium sp.*,* Clostridium sp.*, and* Bacteroides sp.* rapidly become predominant [4]. In naturally delivered, breast-fed children up to 95% of all bacteria are bifidobacteria [8–10] making them by far the predominant bacterial component of the faecal microbiota in this group. The bifidobacteria most frequently isolated from healthy breast-fed infants belong to the species* B. longum*,* B. bifidum*, and* B. breve* [10, 11].

Following the period of exclusive breast-feeding, the composition of the faecal microbiota rapidly changes due to the introduction of solid foods, constant exposure to food-derived and environmental microorganisms, and other factors such as hygiene, antibiotic treatment, and so forth [4, 12]. During the first three years of life, the faecal microbiota then gradually develops into the microbiota of adults [9]. The adult colonic and faecal microbiota is dominated by obligate anaerobes with* Firmicutes* and* Bacteroidetes* together representing more than 80% followed by* Actinobacteria*, which contribute up to 10% to the total bacterial flora. The vast majority (up to 100%) of* Actinobacteria* in faecal samples are representatives of the genus* Bifidobacterium* [12]. Members of this genus are nonmotile, non-spore-forming, strictly anaerobic, gram-positive bacteria characterised by genomes with a high G + C content, an unusual pathway for sugar fermentation termed bifidus shunt, and an unusual V- or Y-shaped morphology formed by most strains under specific culture conditions [13].

### 1.2. Effects of Bifidobacteria on Host Health

In healthy individuals, the composition of the intestinal microbiota is relatively stable throughout adulthood with minor day-to-day variations [36, 37]. However, a number of factors have profound impact on the composition of the microbiota and more substantial and persistent changes in the microbiota, a state also termed dysbiosis, are associated with various diseases [2, 38]. A common feature of most diseases with changes in the (intestinal) microbiota is a reduction or change in the relative abundance of bifidobacteria along with an increase in other bacterial groups, such as* Enterobacteriaceae* or clostridia (Table 1). These alterations might be implicated in onset, perpetuation, and/or progression of disease [12]. However, in most cases, it is not clear whether the altered community profiles of the microbiota are a cause or consequence of the disease.

Besides the implication in various diseases, the intestinal microbiota in general and bifidobacteria in particular are important to establish and maintain health of the host. Studies in germ-free animals nicely illustrate that the presence of a normal microbiota is required for proper development and function of the immune and digestive systems (reviewed in [38, 39]). Their predominance during neonatal development suggests that bifidobacteria play a major role in this process [4].

Various beneficial effects have been claimed to be related to presence or administration of bifidobacteria including cholesterol reduction, improvement of lactose intolerance, alleviation of constipation, and immunomodulation [13, 40, 41]. Different strains of bifidobacteria were shown to have profound effects on dendritic cells, macrophage, and T cells of healthy humans and in animals models of allergy or intestinal inflammation [42–47]. One class of molecules that seems to be of particular relevance for the immunomodulatory properties of bifidobacteria is exopolysaccharides (EPS). Mutants of* B. breve* UCC2003 that lack EPS production induce higher numbers of neutrophils, macrophages, NK, T and B cells in mice compared to the wild type strain indicating that EPS production renders this strain less immunogenic by an unknown mechanism [48].

A promising target for bifidobacterial treatments are amelioration of chronic inflammatory disorders of the GIT [42, 49, 50]. Different strains of bifidobacteria were shown to dampen NF-*κ*B activation and expression and secretion of proinflammatory cytokines by IECs or immune cells in response to challenge with LPS, TNF-*α*, or IL-1*β* [51–56]. Also, various strains of bifidobacteria or mixes of probiotics containing bifidobacteria were able to counteract intestinal inflammation in different models of chronic intestinal inflammation [49, 53, 55–60]. In murine models, different strains of bifidobacteria have been shown to be able to counteract chronic intestinal inflammation by reducing proinflammatory Th1 and inducing regulatory T-cell populations and lowering of colitogenic bacteria [42, 45, 46, 50, 60].

Experiments in mice indicate that some strains of bifidobacteria confer resistance against infections with* Salmonella enterica *serovar Typhimurium [61], enteropathogenic* E. coli* [62, 63], or* Yersinia enterocolitica* [64]. Interestingly,* B. breve* UCC2003 is able to protect mice against infections with* C. rodentium* and this ability depends on EPS production [48, 65]. The protective effect of other bifidobacteria towards enteric infections and intestinal inflammation was shown to be mediated by the production of short chain fatty acids, that is, the end products of bifidobacterial sugar fermentation [50, 63]. It is thus likely that the contribution of EPS production by* B. breve* UCC2003 to protection against* C. rodentium* is related to the improved colonisation [48].

## 2. Colonisation Factors of Bifidobacteria

**Figure 1:**
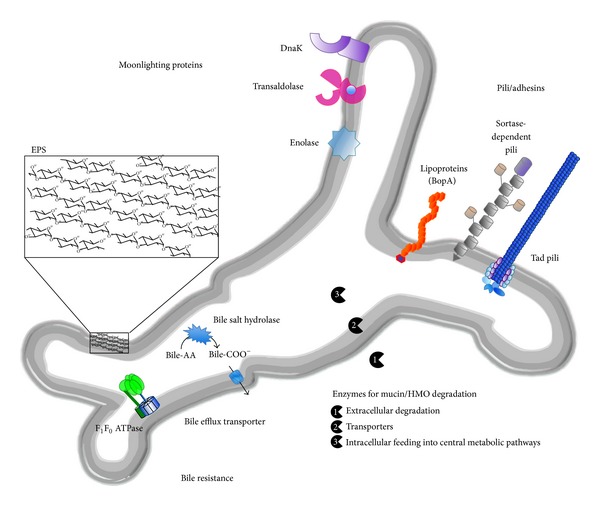
Host colonisation factors of bifidobacteria identified by genome analysis and supported by experimental evidence obtained in* in vitro* experiments and/or murine model systems (bile-AA: conjugated bile acids; bile-COO^−^: deconjugated bile acids; Tad: tight adherence; EPS: exopolysaccharides; HMO: human milk oligosaccharides).

Due to the aforementioned effects of bifidobacteria, genomic approaches were pursued to understand the genetic and physiological traits involved in colonisation of and interaction with the host. The first genome sequence of a* Bifidobacterium sp.* strain was published in 2002 [66]. Since then, the genomes of over 200 strains of bifidobacteria belonging to 25 species and 5 subspecies have been sequenced (http://www.genomesonline.org/). Of these bifidobacterial genomes, 37 are complete and published and 42 are available as permanent drafts. Analysis of these genome sequences has provided insights into the very intimate association of bifidobacteria with their hosts and the adaptation to their gastrointestinal habitat and has led to the identification of a large number of genes with a potential role in these processes [67]. Some of these factors have been analysed in more detail (summarized in Figure 1).

### 2.1. Resistance to Bile

Bile salts are detergents that are synthesized in the liver from cholesterol and secreted via the gall bladder into the GIT lumen [68]. They exert various physiological functions including lipid absorption and cholesterol homeostasis [69]. Since bile salts have considerable antimicrobial activity at physiological concentrations [70], resistance to bile is important for colonisation and persistence of gastrointestinal microorganisms and is thus one of the criteria for the selection of novel probiotic strains [71]. In a number of bifidobacteria, several genes and proteins conferring bile resistance including bile salt hydrolases and bile efflux transporters were identified and characterised* in vitro* [72–82]. Interestingly, the F_1_F_0_-type ATPase of* B. animalis* IPLA4549 was also shown to be involved in bile resistance [83]. The only example for* in vivo* functionality, however, is a recombinant strain of* B. breve* UCC2003 expressing the bile salt hydrolase BilE of* Listeria monocytogenes* [84]. Compared to the wild type, this strain showed improved bile resistance* in vitro* and prolonged gastrointestinal persistence and protection against* L. monocytogenes* infections in mice.

### 2.2. Carbohydrate Utilisation

The genome sequences of bifidobacteria of human origin display a remarkable enrichment in genes involved in breakdown, uptake, and utilisation of a wide variety of complex polysaccharides of dietary and host origin [13, 85–92]. Since most of the simple carbohydrates are absorbed by the host or metabolised by bacteria in the upper gastrointestinal tract, this can be regarded as a specific adaptation of bifidobacteria to their colonic habitat. The ability of bifidobacteria to ferment these complex carbohydrates is the rationale for the use of prebiotics, that is, nondigestible oligosaccharides, to boost bifidobacterial populations in the GIT [93].

The ability to utilise human milk oligosaccharides (HMOs) is thought to provide a selective advantage to bifidobacteria over other microorganisms during initial colonisation of breast-fed newborns and to be, at least partially, responsible for the dominance of bifidobacteria in these children [85, 91]. The genomes of bifidobacteria particularly abundant in breast-fed infants, especially* B. longum* subsp.* infantis*, reflect their adaptation to the utilisation of HMOs [89, 90, 94] and some of the enzymes involved have been characterised [95–97].

Another nutritional adaptation of bifidobacteria to the intestinal niche is the ability to degrade and ferment host-derived mucins. Mucins are high molecular weight glycoproteins secreted by goblet cells as a protective coating for the intestinal epithelium [98]. Similar to the HMO-degradation pathways of* B. longum* subsp.* infantis*,* B. bifidum* strains were shown to grow on mucin as sole carbon source and harbour the respective genes for mucin degradation [85, 92].

### 2.3. Adhesins

Another property frequently associated with host colonisation of commensal and probiotic bacteria is adhesion to intestinal epithelial cells, mucus, or components of the extracellular matrix [99, 100]. Although definite proof for a role of adhesion of bifidobacteria to host-structures in colonisation is missing, these properties are thought to contribute to prolonged persistence and pathogen exclusion. Moreover, the presence of various receptors on the host surface for molecules of probiotic bacteria suggests direct interactions at least at some stage [101].

Strain-dependent adhesion of bifidobacteria to cultured intestinal epithelial cells has been shown in a number of studies [56, 102–115]. However, there are only very few reports investigating adhesion of bifidobacteria from a mechanistic point of view. For example, enolase was shown to mediate binding to human plasminogen by different bifidobacteria [104]. DnaK is another plasminogen-binding protein of* B. animalis* subsp.* lactis* Bl07 [105] and transaldolase is involved in mucus binding of four* B. bifidum* strains [116]. Using a proteomic approach, some of these proteins were shown to be induced in* B. longum* NCC2705 upon cocultivation with intestinal epithelial cells* in vitro* [117]. This indicates that bifidobacteria might be able to sense the presence of intestinal epithelial cells and react by expressing adhesive molecules that mediate interaction with these cells. Interestingly, the role of all these proteins as adhesins seems to be rather a moonlighting function, since they are cytoplasmic proteins with a primary role in bacterial metabolism. Similar moonlighting proteins have been shown to be involved in virulence of different pathogenic bacteria [118].

Bbif_0636, also termed BopA, is a lipoprotein with a cell wall anchor and was previously shown to be involved in adhesion of* B. bifidum* MIMBb75 to IECs [109]. A more detailed analysis performed by our group found the corresponding* bopA* gene to be specifically present in the genomes of* B. bifidum* strains. A purified BopA fusion protein with an N-terminal His_6_-tag inhibited adhesion of* B. bifidum* S17 to IECs. Moreover, expression of this His-tagged protein enhanced adhesion of* B. bifidum* S17 and* B. longum* E18 to IECs. The* bopA* gene is part of an operon encoding a putative oligopeptide ABC transporter and BopA contains an ABC transporter solute-binding domain [109, 112]. This indicates that its primary role might be uptake of nutrients and suggests a moonlighting function in adhesion. A recent study questioned the role of BopA as an adhesin [119]. The authors could show that neither BopA antiserum nor C-terminal His_6_-BopA fusion protein had an effect on adhesion of two* B. bifidum* strains to IECs. However, the His_6_-BopA fusion protein used in this study lacked both the signal sequence and the cell wall anchor motif. Thus, further experiments have to be performed to clarify the role of the position of the His_6_-tag, the contribution of the signal sequence and cell wall anchor, and BopA as an adhesin in general.

A recent bioinformatic analysis of the genome sequence of* B. bifidum* S17 for genetic traits potentially involved in interactions with host tissues revealed that the genome of* B. bifidum* S17 contains at least 10 genes that encode for proteins with domains that have been described or suspected to interact with host tissue components and may thus serve as potential surface-displayed adhesins [120]. Most of the genes for the putative adhesins of* B. bifidum* S17 are expressed* in vitro*, with higher expression during exponential growth phase [120]. Increased expression of the putative adhesins in exponential growth phase was associated with higher adhesion of* B. bifidum* S17 to Caco-2 cells [120].

### 2.4. Pili

All bifidobacterial genomes sequences analysed so far harbour clusters of genes encoding for Tad and/or sortase dependent pili [120–123]. For example,* B. bifidum* S17,* B. breve* S27, and* B. longum* E18 all harbour a complete gene locus for Tad pili. By contrast,* B. longum* E18 genome only contains an incomplete gene cluster for sortase-dependent pili suggesting absence of such structures and* B. breve* S27 encodes one gene cluster and* B. bifidum* S17 encodes three complete gene clusters for sortase-dependent pili. For a range of bifidobacteria, expression of the genes of these pili operons under* in vitro* conditions and in the mouse gastrointestinal tract could be demonstrated [120, 121, 123]. Several studies have also shown presence of pili on the surface of bifidobacteria under these conditions using immunogold labelling and transmission electron microscopy [122] or atomic force microscopy [121, 123]. For one strain of* B. breve* it was demonstrated that Tad pili are indeed important for host colonisation in a murine model [122].

### 2.5. EPS

Genes for EPS production were identified in most genome sequences of* Bifidobacterium sp.* strains [124]. The genetic organisation of EPS gene clusters is not well conserved in bifidobacteria and this is reflected by a high structural variability in the EPS of different bifidobacteria [124]. A recent study has indicated that production of EPS by* B. breve* UCC2003 is important for host colonisation [48]. Mutants of* B. breve* UCC2003 that lack EPS production are significantly less resistant to acidic pH and bile. Moreover, these mutants less efficiently colonize the gastrointestinal tract of mice compared to the wild type strain. Also, EPS-deficient mutants were considerably less immunogenic as the wild type in mice as reflected by lower numbers of immune cells in spleens and lower serum titres of specific antibodies.

Hidalgo-Cantabrana and colleagues characterized the EPS of* B. animalis* subsp.* lactis* A1 and isogenic derivatives, which were obtained by exposure of strain A1 to bile salts (strain A1dOx) followed by cultivation for several generations in the absence of bile (strain A1dOxR). The strain A1dOxR displays a ropy phenotype and shows higher expression of a protein involved in rhamnose biosynthesis along with higher rhamnose content in its EPS [125]. Interestingly, these strains elicited different responses by peripheral blood mononuclear cells and isolated lamina propria immune cells of rats [126].

Despite the presence of EPS gene clusters in most bifidobacteria, it remains to be determined experimentally whether all bifidobacteria actually do produce EPS, if this EPS has a role in host colonisation, and how different EPS structures impact the immune response of the host.

### 2.6. Other Factors Involved in Host Colonisation

Besides bile, another important stress encountered by bifidobacteria during gastrointestinal transit and colonisation is acidic pH in the stomach and small intestine. A number of* B. animalis* subsp.* animalis* and* lactis* strains were shown to survive acidic pH in the physiological range (pH 3–5) in a strain-specific manner and tolerant strains exhibited higher ATPase activity at pH 4 than at pH 5 [127]. Ventura et al. identified the* atp* operon encoding the F_1_F_0_-type ATPase of* B. lactis* DSM10140 and were able to show that its expression was markedly increased upon exposure to acidic pH [128]. Similarly, various ATPase subunits were upregulated in* B. longum* subsp.* longum* NCIMB 8809 in response to acid stress (pH 4.8) as shown by a proteomic approach [129]. This suggests that pH resistance of this strain is inducible and might help to cope with the conditions of the gastrointestinal tract thereby supporting host colonisation. Interestingly, resistance to bile and low pH somehow seems to be connected in the closely related* B. animalis* subsp.* lactis* ILPA 4549. In this strain, expression of the F_1_F_0_-type ATPase and ATPase activity in the membrane was increased in the presence of bile [83]. Moreover, the spontaneous mutant* B. lactis* 4549dOx, which shows increased bile resistance, was also able to better tolerate exposure to acidic pH [83].

More recently, one of the mechanisms by which bifidobacteria might be able to sense their environment and regulate expression of factors important for host colonisation and adaptation to the intestinal niche has been investigated in more detail. A proteomic analysis in* B. longum* NCC2705 identified LuxS as one of the proteins with the most prominent host-induced changes in expression compared to* in vitro* growth [130]. LuxS is an enzyme of the activated methyl cycle of bacteria for recycling of S-adenosylmethionine [131]. By-products of this pathway are autoinducer-2 (AI-2)-like molecules, which are also used by bacteria as signaling molecules and were shown to be involved in biofilm formation, virulence, production of antimicrobials, motility, and genetic competence in a number of gram-positive and gram-negative bacteria [132, 133]. All publicly available genome sequences of bifidobacteria harbour* luxS* homologues, which are functional in the production of AI-2 [134]. Moreover, homologous overexpression of* luxS* in* B. longum* NCC2705 increased AI-2 levels in the supernatant and enhanced biofilm formation [134]. For* B. breve* UCC2003,* luxS* was shown to be important for colonisation of the murine gastrointestinal tract [135].

## 3. Concluding Remarks

Collectively, the available data suggests that individual strains of bifidobacteria exert health-promoting effects on their hosts. An important prerequisite for these effects, is resistance to the conditions of the GIT and, at least, transient colonisation of the host. In recent years, there has been considerable progress in the identification of bifidobacterial structures that play a role in host colonisation and health-promoting effects. However, the vast majority of studies have been performed* in vitro* or in animal models. Based on the fact that they have not been substantiated sufficiently by clinical studies in humans, the European Food Safety Authority has rejected all of the health claims submitted for probiotics. This highlights the need for well-performed clinical trials with a clear definition of target groups and relevant biomarkers and a more detailed analysis of the molecular mechanisms responsible for host colonisation and the positive effects of probiotic bifidobacteria.

## Figures and Tables

**Table 1:** Factors and medical conditions associated with changes in the composition of the faecal microbiota.

Factor/disease	Effect/observation	References
Caesarean section	Higher numbers of the *Clostridium difficile* group l Delayed/reduced colonisation with *Bifidobacterium sp.*, *Lactobacillus sp.* and *Bacteroides sp. *	[14–16]

Infant feeding	Formula-fed infants with lower levels and diversity in *Bifidobacterium sp. *	[11, 15, 17]

Ageing	Increase in *Enterobacteriaceae* and *Bacteroidetes* Reduced levels of *Bifidobacterium sp. *	[18, 19]

Antibiotic-associated diarrhea and chronic *C. difficile* infections	Reduced diversity Increase in *Enterobacteriaceae* and *Firmicutes* Reduced levels of *Bifidobacterium sp.* and *Bacteroidetes *	[18, 20–22]

Irritable bowel syndrome	Increase in *Firmicutes* Reduced levels of *Bacteroidetes* and *Bifidobacterium sp. *	[23–25]

Inflammatory bowel disease	Reduced diversity Lower levels of *Faecalibacterium sp.* Increase in *Enterobacteriaceae* and *Bifidobacterium sp.* Reduced levels of *Bifidobacterium sp.* in pediatric IBD	[26–29]

Atopic disease/Allergy	Increase in *Clostridium sp.* Reduced levels of *Bifidobacterium sp. *	[30–32]

Autism	Increase in *Clostridium sp.* Reduced levels of *Bifidobacterium sp. *	[33–35]
